# Imaging in gynecological disease (29): clinical and ultrasound features of primary ovarian immature teratoma

**DOI:** 10.1002/uog.70111

**Published:** 2025-10-21

**Authors:** C. Landolfo, W. Froyman, A. C. Testa, D. Fischerova, D. Franchi, J. Yazbek, S. Saso, R. Fruscio, V. Chiappa, A. Czekierdowski, T. Van Gorp, F. Moro, L. Savelli, G. F. Zannoni, T. Bourne, D. Timmerman, L. Valentin, Collaborators, S. Guerriero, A. Coosemans, C. Van Holsbeke, A. Sayasneh, S. Helmy‐Bader, L. De Meis, S. Del Forno, R. Heremans, M. Angela Pascual, A. Piras, L. Hovsepyan, A. K. Hiett, J. Y. Verbakel, E. Van Nieuwenhuysen, M. Seckl, I. Vergote

**Affiliations:** ^1^ Queen Charlotte's and Chelsea Hospital Imperial College Healthcare NHS Trust London UK; ^2^ Department of Metabolism, Digestion & Reproduction Faculty of Medicine Imperial College London London UK; ^3^ Department of Development and Regeneration KU Leuven Leuven Belgium; ^4^ Department of Obstetrics and Gynecology University Hospitals Leuven Leuven Belgium; ^5^ Dipartimento Scienze della Salute della Donna, del Bambino e di Sanita' Pubblica Fondazione Policlinico Universitario Agostino Gemelli IRCCS Rome Italy; ^6^ Dipartimento Universitario Scienze della Vita e Sanità Pubblica Università Cattolica del Sacro Cuore Rome Italy; ^7^ Gynecologic Oncology Centre, Department of Gynecology, Obstetrics and Neonatology, First Faculty of Medicine Charles University and General University Hospital in Prague Prague Czech Republic; ^8^ General University Hospital Prague Czech Republic; ^9^ Preventive Gynecology Unit, Division of Gynecology European Institute of Oncology Milan Italy; ^10^ Clinic of Obstetrics and Gynecology University of Milan‐Bicocca, San Gerardo Hospital Monza Italy; ^11^ Department of Gynecologic Oncology Fondazione IRCCS Istituto Nazionale dei Tumori Milan Italy; ^12^ Department of Gynecological Oncology and Gynecology Medical University of Lublin Lublin Poland; ^13^ Department of Oncology, Laboratory of Gynecologic Oncology ImmunOvar Research Group, KU Leuven Leuven Belgium; ^14^ UniCamillus International Medical University Rome Italy; ^15^ Department of Obstetrics and Gynecology Forli and Faenza Hospitals & University of Bologna Bologna Italy; ^16^ Institute of Histopathology Catholic University of the Sacred Heart Rome Italy; ^17^ Department of Obstetrics and Gynecology Skåne University Hospital Malmö Sweden; ^18^ Department of Clinical Sciences Malmö Lund University Malmö Sweden

**Keywords:** ovarian immature teratoma, ovarian neoplasm, ultrasonography

## Abstract

**Objective:**

To describe the clinical and ultrasound characteristics at the time of diagnosis of primary ovarian immature teratoma with no other germ cell tumor components described on histopathology.

**Methods:**

This was a retrospective study of women with a histological diagnosis of primary ovarian immature teratoma who had undergone a preoperative ultrasound examination between 1998 and 2024. Cases were identified from the databases of 17 contributing ultrasound centers and the International Ovarian Tumor Analysis (IOTA) database. The descriptions of the ultrasound images of the tumors made by the original ultrasound examiners using IOTA terminology were reported. In addition, grayscale and color or power Doppler ultrasound images or videoclips were retrieved for all tumors. Two independent ultrasound examiners reviewed the retrieved material and searched for specific ultrasound characteristics of immature teratomas using pattern recognition. We present their agreed description of the tumors.

**Results:**

In total, 64 patients with ovarian immature teratoma were included, of which 38 (59.4%) were obtained from the IOTA database (IOTA studies phase 1, 1b, 2, 3, 5 and 7). The median age of the patients at diagnosis was 24.5 (interquartile range (IQR), 18.8–31.0; range, 12–50) years. The most common presenting symptoms were abdominal or pelvic pain (38/60, 63.3%) and abdominal swelling (30/60, 50.0%). All immature teratomas were unilateral. The median largest diameter of the tumor was 149.5 (IQR, 125.0–183.8; range, 27–400) mm. Using IOTA terminology, most tumors were described as multilocular‐solid (32/64, 50.0%) or solid lesions (22/64, 34.4%). When present, the solid component had a median largest diameter of 98.5 (IQR, 59.8–146.8; range 6–400) mm. Most masses showed minimal (19/63, 30.2%) or moderate (35/63, 55.6%) vascularization on color or power Doppler ultrasound examination. Using pattern recognition, the most typical ultrasound feature was heterogeneous, bizarre echogenicity of the solid components, with hyperechogenic areas, cystic spaces and acoustic shadows. This feature, which we consider pathognomonic, was present in 48/57 (84.2%) immature teratomas in which the solid components were adequately assessable.

**Conclusions:**

The typical ultrasound appearance of an ovarian immature teratoma is a large unilateral adnexal mass with large solid components that is poorly or moderately vascularized. The pathognomonic feature is heterogeneous echogenicity of the solid components with hyperechogenic areas, cystic spaces and acoustic shadows. Preoperative suspicion of immature teratoma can guide treatment, such as offering fertility‐sparing surgery. © 2025 The Author(s). *Ultrasound in Obstetrics & Gynecology* published by John Wiley & Sons Ltd on behalf of International Society of Ultrasound in Obstetrics and Gynecology.

## INTRODUCTION

### Aim

The aim of this study was to describe the clinical and ultrasound characteristics at the time of diagnosis of primary ovarian immature teratoma with no other germ cell tumor components described on histopathology.

### Background

#### Epidemiology

Ovarian immature teratoma is a rare malignant germ cell tumor. It constitutes 3% of all ovarian malignancies and < 1% of all ovarian teratomas^1^, ^2^. It accounts for about a third of all malignant ovarian germ cell tumors^1^. It is most often diagnosed during the first two decades of life and is almost never observed after menopause^3^.

#### Microscopy

Ovarian immature teratoma is composed of a mixture of mature and immature embryonal tissue from ectoderm, mesoderm and endoderm. The presence of immature tissue establishes the diagnosis of immature teratoma, with the most common immature element being neuroepithelium. Other common elements include non‐neural immature epithelium or cartilage. Based on the proportion of the immature neuroectodermal component under any single microscope slide, immature teratomas are histologically graded from Grade 1 to 3^3^. Grade 1 is defined as rare foci of immature neuroectoderm occupying  ≤ 1 low‐power magnification field (40× total magnification, 4.5 mm diameter microscopic field) in any slide; Grade 2 is defined as moderate amounts of immature neuroectoderm occupying > 1 but ≤ 3 low‐power magnification fields in any slide; and Grade 3 is defined as immature neuroectoderm occupying > 3 low‐power magnification fields in any slide^3^. The same grading system is used to describe any metastases of the primary tumor^3^. It is important to assess numerous slices to assign the correct grade. Some pathologists suggest a two‐grade system (low grade *vs* high grade), which combines Grades 2 and 3 into a single high‐grade category^4^, ^5^. Occasionally, immature teratoma is associated with mature (Grade 0) peritoneal implants, so‐called gliomatosis peritonei. Immature teratomas can be found either in pure form or as a component of a mixed malignant germ cell tumor. According to current guidelines, immature teratomas with foci of yolk sac tumor should be reported by pathologists as mixed germ cell tumors^3^. Immature teratomas are classically staged according to the criteria recommended by the International Federation of Gynecology and Obstetrics (FIGO)^3^, ^6^.

#### Macroscopy

In pathology textbooks, ovarian immature teratomas are typically described as unilateral and large. On gross examination they are round, ovoid or lobulated solid masses that may be firm or soft. They appear fleshy and gray‐tan in color and may contain cysts, as well as areas of hemorrhage and necrosis (Figure S1). Occasionally they contain foci of cartilage, bone or hair. The content of the cystic spaces is usually serous but may also be mucinous, colloid or fatty. Immature teratomas do not develop from benign teratomas. However, grossly visible dermoid cysts are found in 26% of immature teratomas, and in 10% of cases there may be a mature cystic teratoma in the contralateral ovary^1^.

#### Clinical presentation and tumor markers

Immature teratomas tend to grow rapidly into large tumors, explaining why patients often present with abdominal pain, distension or bloating. In some cases, the patient may report that she has felt an abdominal mass. Some patients may also present with acute abdominal pain because of ovarian torsion, hemorrhage or rupture of the lesion^1^. Rarely, the diagnosis is made in a completely asymptomatic patient^2^.

There is no tumor marker specific to immature teratoma. Serum levels of lactate dehydrogenase and human chorionic gonadotropin are usually normal in patients with immature teratoma. Serum levels of alpha‐fetoprotein (AFP) may be elevated both in pure immature teratomas and in immature teratomas with foci of yolk sac tumor, even in those with microscopic foci (≤ 3 mm) of yolk sac tumor (Heifetz lesions)^5^. Elevated serum levels of AFP in patients with immature teratoma may be explained by immature liver and gastrointestinal components and not primarily by foci of yolk sac tumor^7^. Another explanation for raised serum levels of AFP in patients with immature teratoma without histologically documented yolk sac tumor elements could be sampling error because immature teratomas are usually large and therefore small areas of yolk sac tumor may be missed.

#### Management and prognosis

The prognosis for patients treated for ovarian immature teratoma is favorable. The 5‐year overall survival rate is > 90%, and for Stage‐I tumors this 5‐year survival rate approaches 100%^3^. The stage and grade of the primary tumor and the grade of metastases, if present, are the main prognostic factors and are important for informing choice of treatment^3^, ^5^. Even though gliomatosis peritonei classifies the tumor as FIGO Stage III, its behavior is generally benign^3^.

Because of the rarity of immature teratoma, patients with this tumor should be treated in a large cancer center^5^. Most patients with immature teratoma are young at the time of diagnosis; therefore, fertility‐sparing surgery is now recommended for patients who want to preserve their fertility, even though data are scarce for Grade 2 and 3 Stage II–IV immature teratoma^8^. Close and active surveillance without adjuvant chemotherapy is recommended for patients with Grade‐1 Stage‐IA immature teratoma^5^. Adjuvant chemotherapy for patients with Grade‐2 or Grade‐3 Stage‐IA immature teratoma and Stage IB–IC is controversial^5^. The standard of care for adult women with Stage‐II–IV ovarian immature teratoma is postoperative chemotherapy, while close surveillance alone is often recommended for pediatric patients irrespective of grade and stage^5^, ^9^. Immature teratomas exhibit sensitivity to chemotherapy, usually including bleomycin, etoposide and platinum^9^, ^10^, ^11^. In case of chemoresistance and relapse, surgical management should be considered, especially if complete resection is achievable^5^.

Growing teratoma syndrome is a rare condition that may occur in patients with a history of immature teratoma treated with chemotherapy^5^, ^12^, ^13^. In this condition, growing benign mature teratoma masses are diagnosed during or after chemotherapy, despite normalization of serum levels of AFP, if previously elevated^14^. Although these growing teratoma masses are benign, they can cause severe complications by compressing surrounding organs. Growing teratoma syndrome is therefore treated with surgery. Long‐time follow‐up is needed due to the risk of late recurrence^15^.

## METHODS

This was an international retrospective multicenter study. From the databases of 17 contributing ultrasound centers and from the International Ovarian Tumor Analysis (IOTA) database, we identified patients with a histologically confirmed diagnosis of primary ovarian immature teratoma between 1998 and 2024, in which no other malignant germ cell components were described on histology. All patients included in the study had undergone ultrasound examination prior to surgical treatment. Relapses and mixed germ cell tumors with immature teratoma components were not included. The contributing centers are listed in Appendix S1. Patients included in IOTA studies phase 1, 1b, 2, 3 and 5^16^, ^17^, ^18^, ^19^, ^20^, as well as the ongoing IOTA 7 study (ClinicalTrials.gov: NCT02847832), had been examined using ultrasound following a strict research protocol, employing IOTA standardized examination and measurement techniques and IOTA terminology for describing the ultrasound images^21^. For these patients, ultrasound features and predefined clinical information had been prospectively entered into the IOTA database. For patients identified outside of the IOTA studies, one expert ultrasound examiner from each center retrospectively reviewed stored ultrasound reports and images and described the tumor using IOTA terminology^21^. Information was then entered into a dedicated Excel file (version 16.90; Microsoft Corp., Redmond, WA, USA).

To be included in the present study, patients needed to have undergone both grayscale and color or power Doppler ultrasound examination, and there had to be sufficient documentation to allow description of the tumor using IOTA terminology, including the assignment of an IOTA color score according to vascularization of the tumor: no detectable blood flow (color score = 1), minimal blood flow (color score = 2), moderate blood flow (color score = 3) or abundant blood flow (color score = 4). For all patients examined outside of the IOTA studies, and in case of missing clinical information in the IOTA database (such as tumor markers and symptoms), information was retrospectively retrieved from patient records. For all cases, final histology, grade and FIGO stage were defined by the local pathologist.

All ultrasound examinations had been performed ≤ 120 days before surgical removal of the mass, with all patients undergoing transvaginal ultrasound, supplemented with a transabdominal scan when necessary. The examinations were carried out using mid‐range or high‐end ultrasound equipment: Voluson E6, Voluson E8 or Voluson E10 (GE Healthcare, Zipf, Austria); Samsung WS80A, Samsung HS70A, Samsung HS60 or Samsung Hera I10 (Samsung Medison Co. Ltd., Seoul, South Korea); Esaote MyLab 70 (Esaote S.p.A., Genoa, Italy); ATL HDI 5000 (Philips, Amsterdam, The Netherlands); Sequoia 512 (Acuson Inc., Mountain View, CA, USA); or Siemens S2000 (Siemens Healthineers, Malvern, PA, USA). The diagnosis suggested by the original ultrasound examiner (whether benign or malignant, and the specific histological type) as registered in the IOTA database, or in the original ultrasound report if the patient was not included in an IOTA study, was recorded.

In addition to describing the immature teratomas based on the information in the IOTA database and in patient records or ultrasound reports, two authors (C.L., L.V.) reviewed all available ultrasound images and videoclips. They described these independently using subjective evaluation of grayscale and color or power Doppler ultrasound images to try and identify possible characteristic ultrasound features through pattern recognition^22^. Discrepancies were resolved through discussion until consensus was reached. We report the description agreed between the two authors.

We present results as absolute numbers and percentages for nominal variables and as median (interquartile range (IQR); range) for continuous variables.

## RESULTS

In total, 64 patients with ovarian immature teratoma were identified. Thirty‐eight (59.4%) patients were identified from the IOTA database (one from IOTA phase 1^16^, one from IOTA phase 1b^17^, one from IOTA phase 2^18^, six from IOTA phase 3^19^, six from IOTA phase 5^20^ and 23 from the ongoing IOTA phase 7 study), while 26 patients had not been included in any IOTA study.

Demographic background data, tumor grade and FIGO stage are shown in Table 1. The median age at diagnosis was 24.5 (IQR, 18.8–31.0; range, 12–50) years. Only three patients were older than 40 years. All patients were premenopausal and two were pregnant when the diagnosis was made. The most common presenting symptoms were abdominal or pelvic pain (38/60, 63.3%) and abdominal swelling (30/60, 50.0%). Six (10.0%) patients were asymptomatic. When assessed, serum cancer antigen 125 (CA125) levels were raised (> 35 IU/mL) in 39/55 (70.9%) patients and serum AFP levels were raised (> 14 ng/mL) in 22/34 (64.7%) patients. Most immature teratomas were high grade (i.e. Grade 2 or 3) (38/57, 66.7%) and FIGO Stage I (53/64, 82.8%).

**Table 1:** Clinical and tumor characteristics of 64 patients with primary ovarian immature teratoma

Characteristic	Value
Age at diagnosis (years)	24.5 (18.8–31.0) [12.0–50.0]
Premenopausal	64 (100)
Nulliparous	40/58 (69.0)
Pregnant at diagnosis	2 (3.1)
Previous gynecological surgery	
Hysterectomy	0/59 (0)
Unilateral salpingo‐oophorectomy	7/59 (11.9)
Tumor marker serum levels at diagnosis	
CA125 (IU/mL)*	83 (29.0–190.0) [1.0–549.0]
AFP (ng/mL)†	49.0 (10.0–413.0) [0.5–3611.0]
CEA (ng/mL)‡	3.4 (1.6–6.2) [0.3–10.0]
CA19‐9 (IU/mL)§	37.0 (14.2–161.8) [1.0–807.0]
hCG (IU/mL)¶	1.1 (0.1–2.0) [0.1–107.0]
Grade of tumor	
1 (low grade)	19/57 (33.3)
2 or 3 (high grade)	38/57 (66.7)
FIGO Stage	
I	53 (82.8)
II	2 (3.1)
III	9 (14.1)
IV	0 (0)

Data are given as median (interquartile range) [range], *n* (%) or *n*/*N* (%).

* Information available for 55 (85.9%) cases.

† Information available for 34 (53.1%) cases. In three patients, serum level of alpha‐fetoprotein (AFP) was > 1000 ng/mL (1605 ng/mL in a Grade‐3 Stage‐IC tumor, 1769 ng/mL in a Grade‐2 Stage‐IA tumor and 3611 ng/mL in a Grade‐3 Stage‐IA tumor); in all three cases, the pathologist made a diagnosis of immature teratoma and did not mention the presence of any other germ cell tumor elements in the histological report.

‡ Information available for 27 (42.2%) cases.

§ Information available for 30 (46.9%) cases.

¶ Information available for 22 (34.4%) cases. CA, cancer antigen; CEA, carcinoembryonic antigen; FIGO, International Federation of Gynecology and Obstetrics; hCG, human chorionic gonadotropin.

### Ultrasound characteristics as described by original ultrasound examiner

The ultrasound characteristics of the immature teratomas, as described by the original ultrasound examiner in the IOTA database or in the patient records, are presented in Table 2. All tumors were unilateral. The immature teratomas were large, with the median largest diameter being 149.5 (IQR, 125.0–183.8) mm. Only one tumor was smaller than 65 mm, and 58/64 (90.6%) tumors were larger than 100 mm. All but three tumors contained solid components (61/64, 95.3%). When present, the solid components were large, with a median largest diameter of 98.5 (IQR, 59.8–146.8) mm. Most lesions were described as multilocular‐solid (32/64, 50.0%) or solid (22/64, 34.4%), and most immature teratomas showed minimal (color score of 2) (19/63, 30.2%) or moderate (color score of 3) (35/63, 55.6%) vascularization on color or power Doppler examination. Ascites was described in 9/64 (14.1%) patients. In 55/64 (85.9%) patients, the original ultrasound examiner classified the mass as borderline or invasive malignant.

**Table 2:** Ultrasound (US) characteristics of 64 ovarian immature teratomas and diagnosis suggested by the original examiner, as described in the International Ovarian Tumor Analysis (IOTA) database or original US reports

US characteristic or diagnosis	Value
Unilateral tumor	64 (100)
Ascites	9 (14.1)
Free fluid in pouch of Douglas	23/63 (36.5)
Largest diameter of lesion (mm)	149.5 (125.0–183.8) [27.0–400.0]
Type of tumor	
Unilocular	0 (0)
Unilocular‐solid	7 (10.9)
Multilocular	3 (4.7)
Multilocular‐solid	32 (50.0)
Solid	22 (34.4)
Number of locules for multilocular and multilocular‐solid tumors	
≤ 3	4/34 (11.8)
4–5	10/34 (29.4)
6–10	6/34 (17.6)
> 10	14/34 (41.2)
Echogenicity of cyst fluid in tumors not classified as solid	
Anechoic	14/42 (33.3)
Low level	18/42 (42.9)
Mixed	10/42 (23.8)
Largest diameter of largest solid component (mm)*	98.5 (59.8–146.8) [6.0–400.0]
Presence of papillary projections	8 (12.5)
Number of papillary projections	
1	4/8 (50.0)
2	1/8 (12.5)
3	2/8 (25.0)
> 3	1/8 (12.5)
Height of largest papillary projection (mm)	30 (18.0–53.3) [5.0–67.0]
Vascularized papillary projection	6/8 (75.0)
Irregular cyst walls	50 (78.1)
Incomplete septum	2/57 (3.5)
Shadowing	41 (64.1)
Doppler color score	
1 (no vascularization)	3/63 (4.8)
2 (minimal vascularization)	19/63 (30.2)
3 (moderate vascularization)	35/63 (55.6)
4 (abundant vascularization)	6/63 (9.5)
Diagnosis based on subjective assessment by original US examiner	
Benign	9 (14.1)
Borderline or malignant	55 (85.9)
Specific diagnosis suggested by original US examiner	
Malignant rare tumor	19 (29.7)
Immature teratoma	12 (18.8)
Primary ovarian cancer	10 (15.6)
Benign mature teratoma	9 (14.1)
Borderline tumor	5 (7.8)
Germ cell tumor	4 (6.3)
Not possible	4 (6.3)
Low‐grade ovarian cancer	1 (1.6)

Data are given as *n* (%), *n*/*N* (%) or median (interquartile range) [range].

* Information available for 61/64 (95.3%) cases. IOTA terminology is used^21^.

### Ultrasound characteristics based on pattern recognition

**Figure 1:**
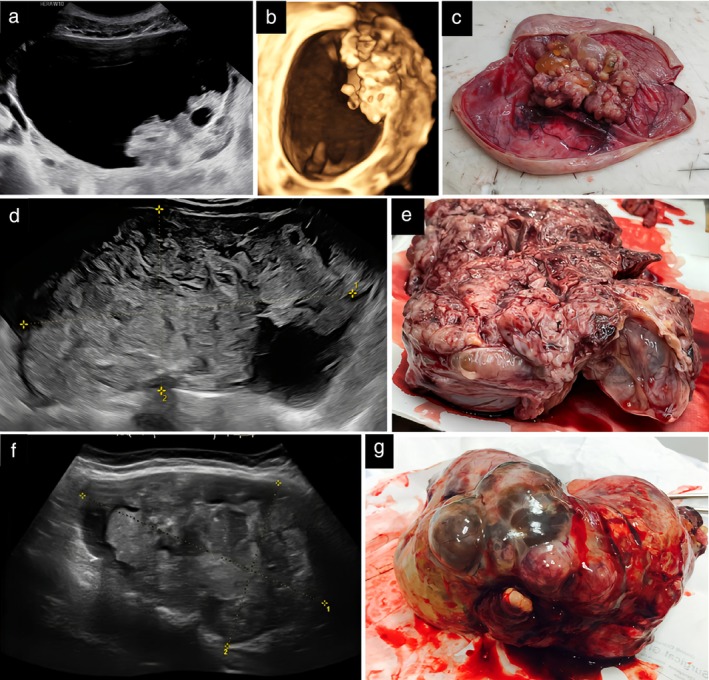
Grayscale ultrasound images (a,d,f), three‐dimensional rendering (b) and corresponding macroscopic appearance (c,e,g) of three ovarian immature teratomas: (a–c) tumor 1, (d,e) tumor 2 and (f,g) tumor 3.

**Figure 2:**
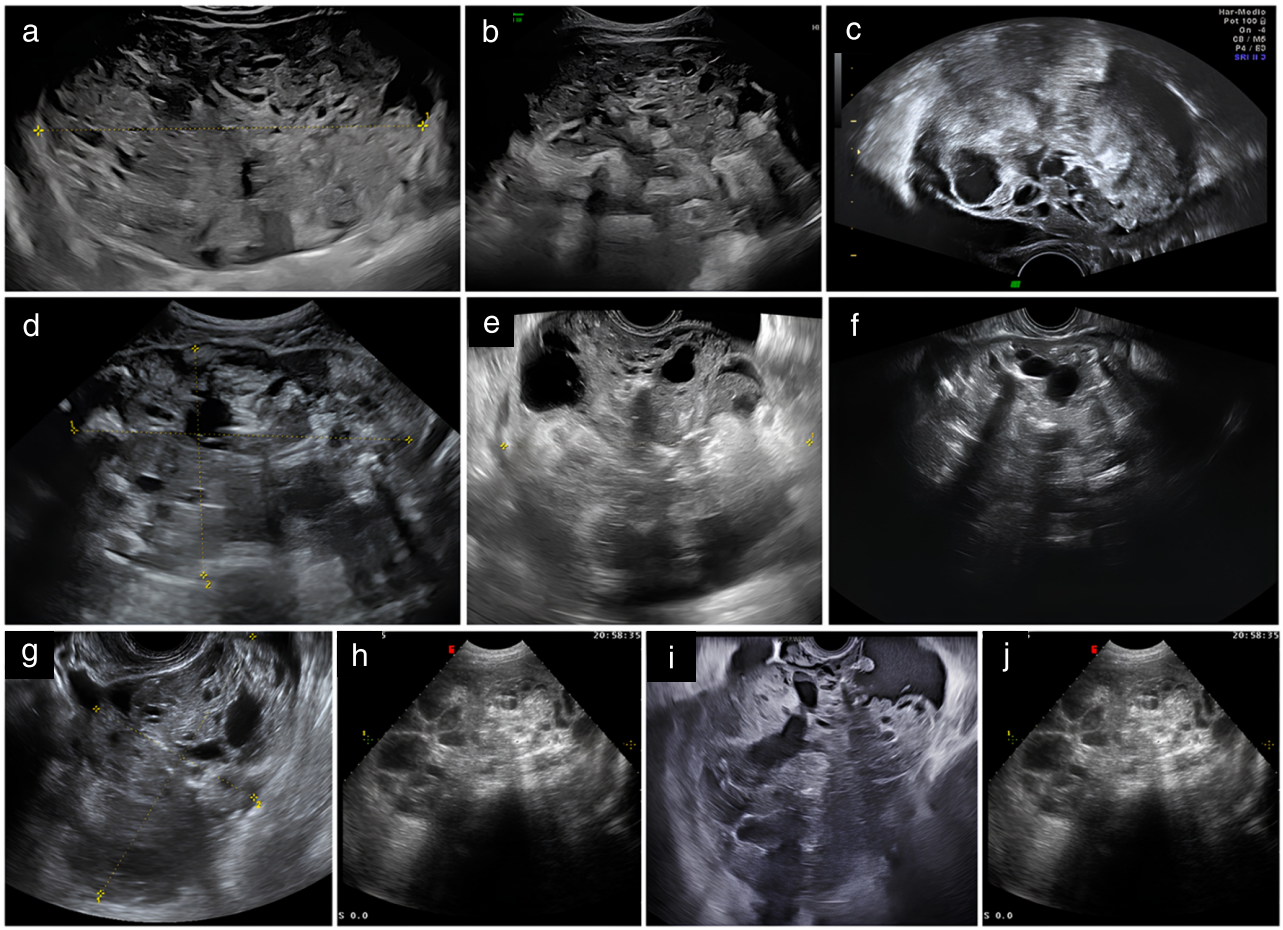
(a–j) Grayscale ultrasound images of 10 ovarian immature teratomas. Using pattern recognition, these tumors were described as solid tumors (lesions consisting of ≥ 80% solid tissue). They all show pathognomonic echogenicity of solid tissue, with hyperechogenic areas, cysts and shadowing.

**Figure 3:**
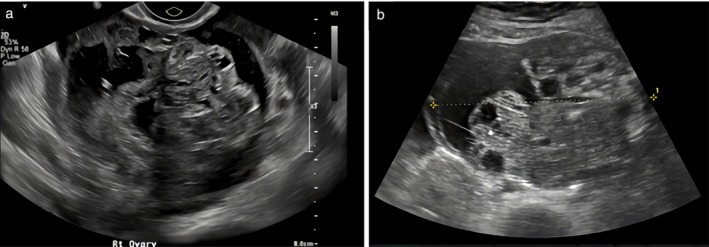
(a,b) Grayscale ultrasound images of two pure immature teratomas of the ovary. Using pattern recognition, these tumors were described as cystic tumors with solid components that seemed to arise from the cyst wall, but did not protrude into the cyst cavity like a papillary projection would. Solid components show pathognomonic echogenicity, with hyperechogenic areas, cysts and shadowing.

**Figure 4:**
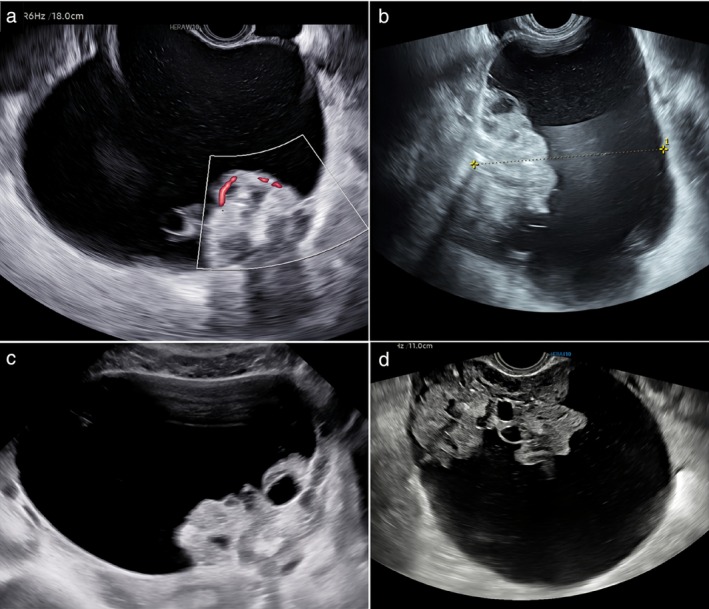
Color Doppler (a) and grayscale (b,c,d) ultrasound images of four ovarian immature teratomas. Using pattern recognition, these tumors were described as cystic with a solid component protruding into the cyst cavity like a large papillary projection. Solid components show pathognomonic echogenicity, with hyperechogenic areas, cysts and shadowing.

**Figure 5:**
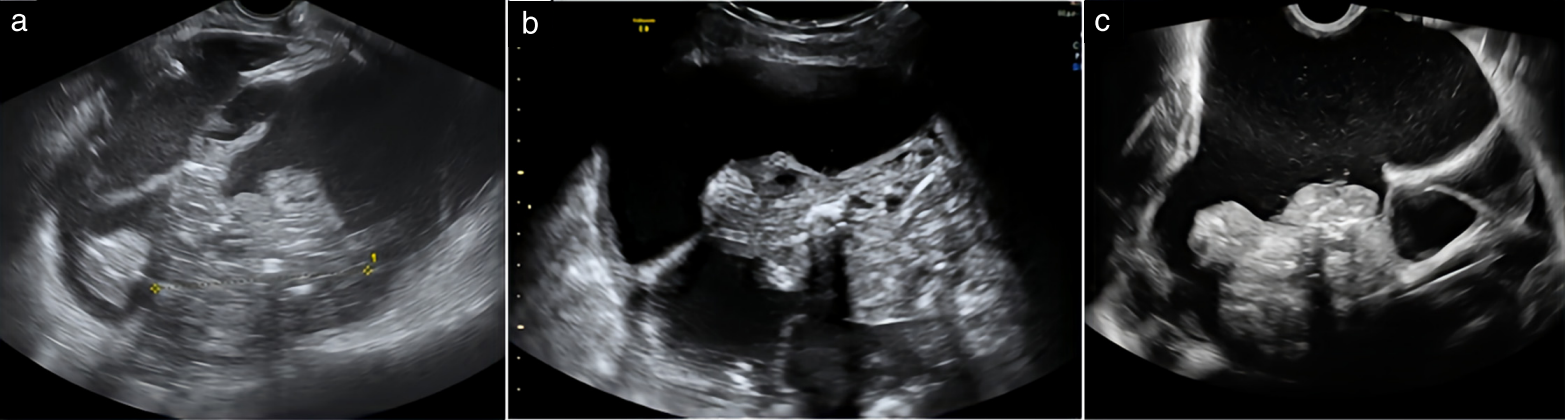
(a–c) Grayscale ultrasound images of three ovarian immature teratomas. Using pattern recognition, these tumors were described as multilocular‐solid tumors with solid tissue entrapped among septa. Solid components show pathognomonic echogenicity, with hyperechogenic areas, cysts and shadowing.

**Figure 6:**
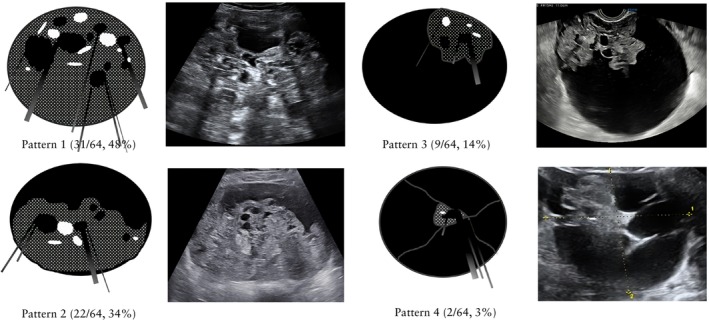
Schematic drawing of the four identified ultrasound patterns of immature teratomas. Pattern 1: a purely solid tumor, i.e. a tumor consisting of ≥ 80% solid tissue; Pattern 2: a cystic tumor with a solid component that seemed to arise from the cyst wall but did not protrude into the cyst cavity like a papillary projection would; Pattern 3: a cystic tumor with a solid component that seemed to protrude into the cyst cavity like a large papillary projection; and Pattern 4: a multilocular‐solid tumor with solid tissue entrapped among septa. The stippled area in the schematic drawings represents the solid component, the black areas represent the cystic components, the white areas represent the hyperechogenic areas and the black and gray lines represent the shadows in the corresponding ultrasound image.

Grayscale images, with or without color or power Doppler ultrasound images, were available for all 64 tumors (paper images for four tumors and electronically stored images or videoclips for the remaining 60 tumors). For nine tumors, we also had access to the corresponding macroscopic appearance of the surgical specimen (three examples are shown in Figure 1). Using pattern recognition, we identified four patterns: (1) a purely solid tumor, i.e. a tumor consisting of ≥ 80% solid tissue (Figure 2); (2) a cystic tumor with a solid component that seemed to arise from the cyst wall but did not protrude into the cyst cavity like a papillary projection would (Figure 3); (3) a cystic tumor with a solid component that seemed to protrude into the cyst cavity like a large papillary projection (Figure 4); and (4) a multilocular‐solid tumor with solid tissue entrapped among septa (Figure 5). A schematic drawing of the four patterns is shown in Figure 6. The purely solid pattern (pattern 1) was the most common (31/64, 48.4%), followed by pattern 2 (22/64, 34.4%). Patterns 3 and 4 were observed in 9/64 (14.1%) and 2/64 (3.1%) tumors, respectively.

**Figure 7:**
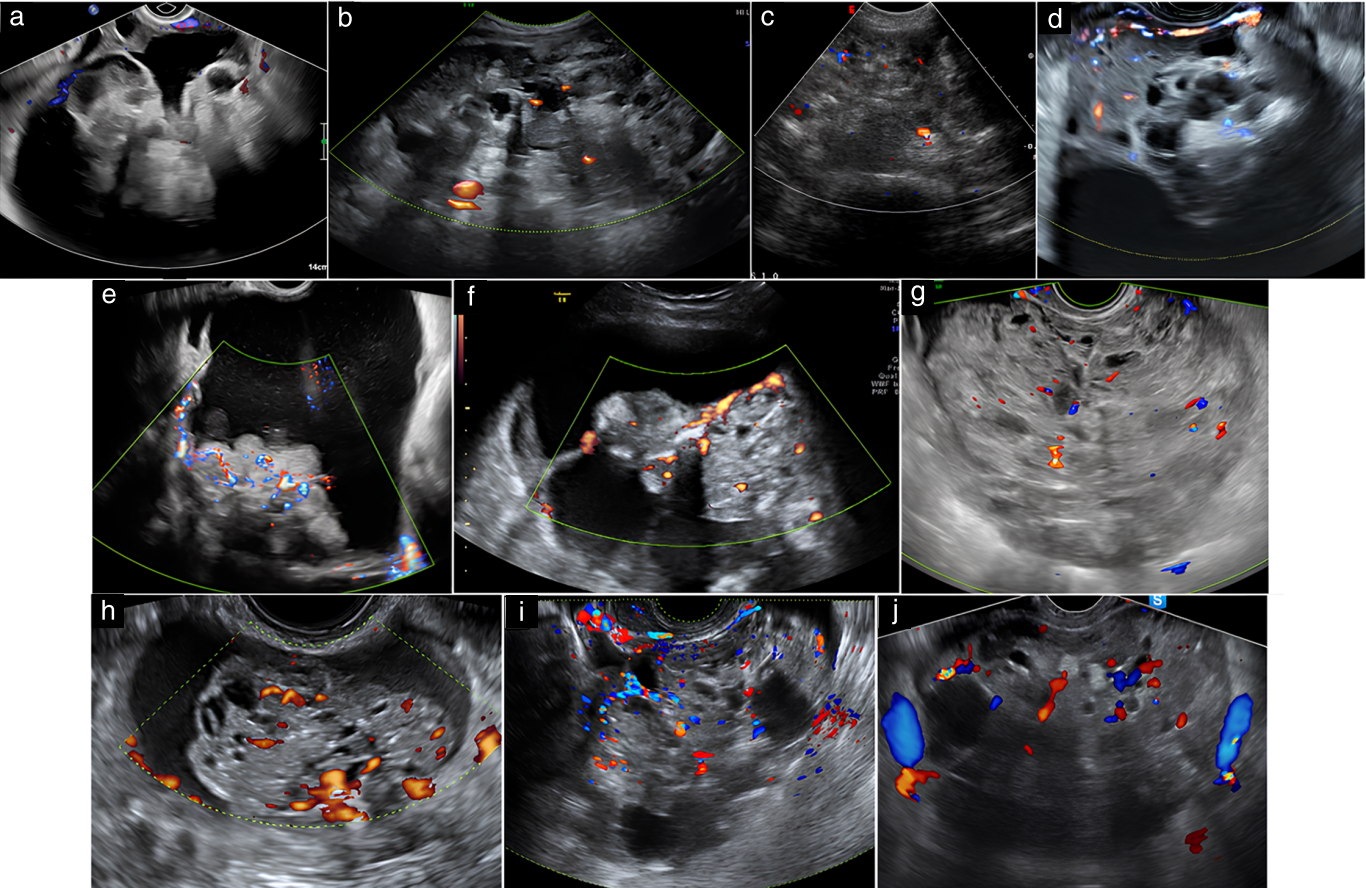
Color (a,c,d,e,g,i,j) and power (b,f,h) Doppler ultrasound images of 10 ovarian immature teratomas showing minimal (a–c) or moderate (d–j) vascularization. Pulse repetition frequency was 0.3 or 0.6 kHz.

A common characteristic of the immature teratomas was heterogeneous, bizarre echogenicity of the solid tissue with hyperechogenic areas, cystic spaces and acoustic shadowing (Figures 2, 3, 4, 5). Using pattern recognition, the echogenicity of the solid components could be properly assessed in 57 tumors. In seven tumors, the solid component was either too small or too far away from the ultrasound probe, or the image quality was too poor to allow detailed assessment of the echogenicity of the solid component (Figure S2). The characteristic echogenicity of an immature teratoma (hyperechogenic areas, cystic spaces and shadowing) was found in 48/57 (84.2%) tumors. The other nine tumors (9/57, 15.8%) did not manifest this pathognomonic echogenicity in their solid components (Figure S3). Based on pattern recognition, most tumors showed acoustic shadows (59/64, 92.2%) and poor or moderate vascularization on color or power Doppler (49/52, 94.2%; the available color or power Doppler images were of sufficient quality for reliable assessment of the color content for only 52 tumors) (Figure 7). Two tumors did not manifest any of the typical ultrasound features of an immature teratoma. Their largest diameter was 27 mm and 65 mm, respectively, and normal ovarian tissue was visible adjacent to the tumor (Figure S4). In both cases, the patient had a personal history of a surgically removed malignant ovarian tumor in the contralateral ovary (serous borderline tumor and yolk sac tumor, respectively).

## DISCUSSION

Herein, we have described the clinical and ultrasound characteristics of primary ovarian immature teratoma. We found most ovarian immature teratomas were diagnosed in patients ≤ 30 years old and presented as large unilateral tumors with large solid components that were poorly or moderately vascularized on color or power Doppler ultrasound examination. Based on pattern recognition, the most typical ultrasound feature was heterogeneous, bizarre echogenicity of the solid components with hyperechogenic areas, cystic spaces and acoustic shadows. We consider this echogenicity pathognomonic of an immature teratoma.

To the best of our knowledge, this is the largest study to date describing the ultrasound features of ovarian immature teratoma. Another strength is the use of the standardized IOTA terminology to describe the ultrasound images. The main limitation is that the study is retrospective, which means that some information was incomplete, such as symptoms and tumor markers. Moreover, for some of the tumors, only few or suboptimal ultrasound images and videoclips were available. Another possible limitation is that most tumors in our series were diagnosed in referral centers for ultrasonography. This means that the immature teratomas in our study may be a selection of tumors with more complex ultrasound morphology than immature teratomas detected and treated in non‐referral centers. It is also a limitation that the two reviewers of the ultrasound images were aware of the histology of each tumor, which could have introduced bias when interpreting the images. In addition, the images and videoclips were collected over a 25‐year period. Image quality changed over time, and this may have affected the results of pattern recognition. Finally, it is a limitation that we did not perform a central pathology review because the histological diagnosis is somewhat subjective regarding the amount of immature neuroectodermal tissue. In some patients with raised serum levels of AFP, yolk sac tumor elements may have been missed by the pathologist, even though the pathologists classified all tumors included in this series as an immature teratoma and did not report the presence of yolk sac elements in the tumor.

In an extensive literature search, we found only two publications describing the ultrasound appearance of immature teratomas^23^, ^24^. The review article of Sutton *et al*.^23^ includes one immature teratoma and presents one abdominal ultrasound image, showing the tumor with large solid components and cystic spaces. The image quality is low compared with contemporary ultrasound images, but there is some resemblance to our images of immature teratomas, with the large solid tumor components being hyperechogenic. Bazot *et al*.^24^ describe two cases of ‘dermoid cysts with foci of immature tissue’. The published ultrasound images of these show cystic tumors with a large solid component protruding into the cyst cavity like a large papillary projection and manifesting the typical heterogenous echogenicity of an immature teratoma with hyperechogenic solid tissue, cystic spaces and acoustic shadowing. Saba *et al*.^25^ describe and show computed tomography (CT) and magnetic resonance (MRI) images, but no ultrasound images, of ovarian immature teratomas. They describe the solid components of immature teratomas as large and irregular with calcifications and small foci of fat and hemorrhage.

The two reviewers of the ultrasound images and videoclips in our study found the echogenicity of the solid tissue in immature teratomas to be pathognomonic and noted that the sonographic appearance of immature teratomas was completely different from that of benign dermoid cysts (Figure S5)^26^. The typical heterogeneous echogenicity of the solid tumor components of an immature teratoma reflects their typical macroscopic appearance, which in turn is explained by the mixture of different types of undifferentiated embryonal tissue (Figures 1 and S1). Therefore, in typical cases, it should be possible to suggest a diagnosis of immature teratoma preoperatively based on ultrasound characteristics and clinical information, such as young age. However, the differential diagnosis between benign cystic teratoma and immature teratoma is sometimes challenging; for example, in large cystic tumors with small solid components, or when solid components are located far from the ultrasound probe, detailed evaluation of the echogenicity of these components can be difficult. Benign cystic teratomas with vascularized solid components and other non‐typical benign teratomas can also pose a diagnostic dilemma (Figure S6)^26^.

It is clinically relevant to be able to suspect ovarian immature teratoma preoperatively. Because ovarian immature teratoma is a very rare tumor, patients in whom it is suspected should be referred to a large cancer center for management. Serum levels of malignant germ cell tumor markers should be measured and whole‐body MRI (preferable to CT scan in young patients) should be performed to estimate tumor spread. Moreover, a preoperative suspicion of ovarian immature teratoma can guide treatment, such as offering fertility‐sparing surgery.

Now that the typical ultrasound features of ovarian immature teratoma have been described, future prospective studies should validate the ability of ultrasound examiners to correctly predict a postoperative diagnosis of immature teratoma. The ability of artificial intelligence to recognize an immature teratoma on ultrasound could also be explored.

In conclusion, an ultrasound finding of a unilateral large adnexal mass with large solid components manifesting hyperechogenic areas, cystic spaces and acoustic shadowing in a young patient should raise a suspicion of ovarian immature teratoma.

## Collaborators

**S. Guerriero**, Centro Integrato di Procreazione Medicalmente Assistita (PMA) e Diagnostica Ostetrico‐Ginecologica, Azienda Ospedaliero Universitaria Cagliari‐Policlinico Duilio Casula, Monserrato, Italy and Department of Surgical Sciences, University of Cagliari, Cagliari, Italy

**A. Coosemans**, Department of Oncology, Laboratory of Tumor Immunology and Immunotherapy, Leuven Cancer Institute, KU Leuven, Leuven, Belgium

**C. Van Holsbeke**, Department of Obstetrics and Gynecology, Ziekenhuis Oost‐Limburg, Genk, Belgium

**A. Sayasneh**, Guy's Campus, School of Life Course Sciences, Faculty of Life Sciences and Medicine, King's College London, London, UK

**S. Helmy‐Bader**, Department of General Gynecology and Gynecologic Oncology, Vienna, Austria

**L. De Meis**, Gynecology and Physiopathology of Human Reproduction Unit, S. Orsola‐Malpighi Hospital of Bologna, Bologna, Italy

**S. Del Forno**, Gynecology and Physiopathology of Human Reproduction Unit, S. Orsola‐Malpighi Hospital of Bologna, Bologna, Italy

**R. Heremans**, Department of Development and Regeneration, KU Leuven, Leuven, Belgium and Department of Obstetrics and Gynecology, University Hospitals Leuven, Leuven, Belgium

**M. Angela Pascual**, Department of Obstetrics, Gynecology, and Reproduction, Hospital Universitari Dexeus, Barcelona, Spain

**A. Piras**, Department of Obstetrics and Gynecology, Azienda Ospedaliero Universitaria di Cagliari, Cagliari, Italy

**L. Hovsepyan**, Department of Obstetrics and Gynecology, Erebuni Medical Center, Yerevan, Armenia

**A. K. Hiett**, Department of Obstetrics and Gynecology, Wright State University, Boonshoft School of Medicine, Dayton, OH, USA

**J. Y. Verbakel**, Department of Public Health and Primary Care, KU Leuven, Leuven, Belgium

**E. Van Nieuwenhuysen**, Department of Obstetrics and Gynecology, University Hospitals Leuven, Leuven, Belgium and Department of Development and Regeneration, KU Leuven, Leuven, Belgium

**M. Seckl**, Department of Medical Oncology, Charing Cross Hospital Campus of Imperial College London, London, UK

**I. Vergote**, Department of Obstetrics and Gynecology, University Hospitals Leuven, Leuven, Belgium

## Supporting information


**Figure S1** Macroscopic image of immature teratoma of the ovary with solid fleshy consistency and multiple small yellowish cysts.

**Figure S2** Grayscale (a,b) and color Doppler (c–e) ultrasound images of five ovarian immature teratomas in which the solid components were small in comparison to the whole tumor. In (b–e), the solid component was too far away from the ultrasound probe for detailed assessment of its echogenicity to be possible.

**Figure S3** Grayscale (a–c) and color Doppler (d) ultrasound images of four ovarian immature teratomas in which large solid components did not manifest pathognomonic echogenicity with hyperechogenic areas, cystic areas and shadowing.

**Figure S4** Grayscale (a–c) and color Doppler (d) ultrasound images of two ovarian immature teratomas that did not manifest typical ultrasound features of immature teratoma. Both Stage IIA immature teratoma (largest diameter, 27 mm) (a,b) and Grade 1 Stage IC immature teratoma (largest diameter, 65 mm) (c,d) manifest normal ovarian parenchyma visible adjacent to the tumor (white arrow). Both patients had a personal history of a surgically removed malignant ovarian tumor in the contralateral ovary (borderline tumor (a,b) and yolk sac tumor (c,d), respectively).

**Figure S5** Typical ultrasound images of immature teratomas with pathognomonic appearance of the solid components (i.e. cystic areas, hyperechogenic areas and shadowing) (a–d), contrasted with typical ultrasound images of dermoid cysts (e–h) which display features resembling ‘white ball’ (e), ‘dots and lines’ (f), ‘cotton wool tufts’ (g) and ‘mushroom cap’ (h). The ultrasound images of the dermoid cysts are reproduced from Heremans *et al*.^26^.

**Figure S6** Color (a) and power (b) Doppler ultrasound images of benign cystic teratoma with atypical ultrasound appearance (a) and an immature teratoma with only a small solid component (b). The ultrasound image of the benign cystic teratoma is reproduced from Heremans *et al*.^26^.

**Appendix S1** List of contributing ultrasound centers.

## Data Availability

The data that support the findings of this study are available from the corresponding author upon reasonable request.
