# Supplementary figures and images for: Imaging in gynecological disease (29): clinical and ultrasound features of primary ovarian immature teratoma
Source: Ultrasound Obstet Gynecol. 2025 Oct 21;67(1):89–99. doi: 10.1002/uog.70111 (PMC12757824; doi:10.1002/uog.70111)

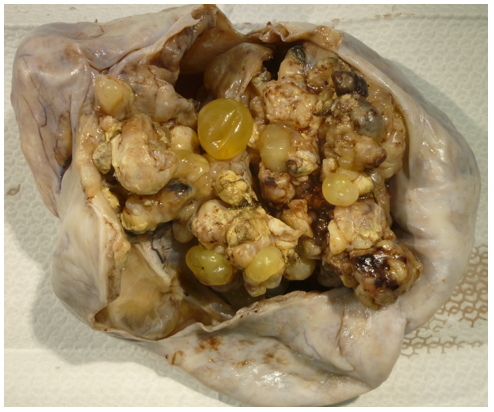

Supplement: Supplementary file 1 — Figure S1 Macroscopic image of immature teratoma of the ovary with solid fleshy consistency and multiple small yellowish cysts. [file UOG-67-89-s005.png]

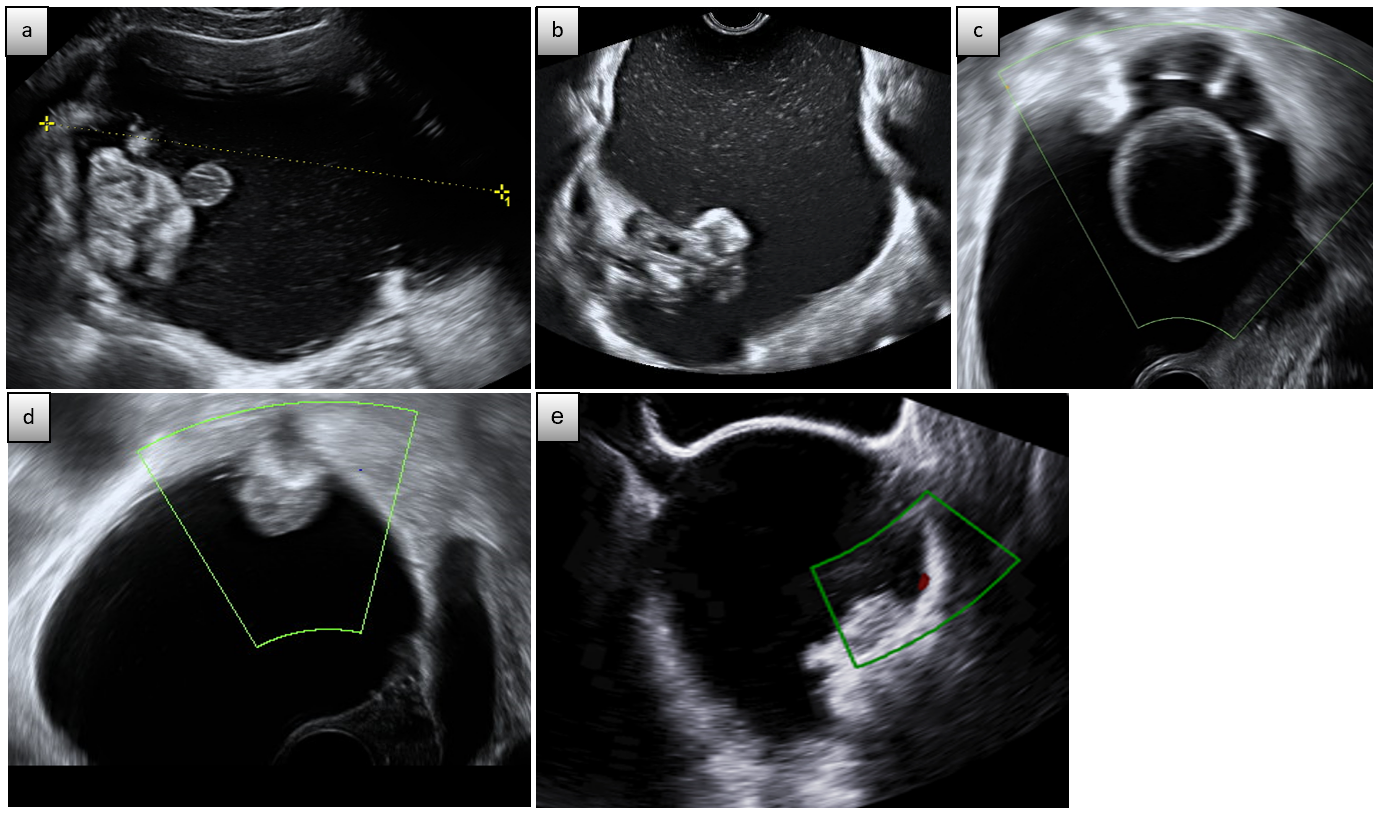

Supplement: Supplementary file 2 — Figure S2 Grayscale (a,b) and color Doppler (c–e) ultrasound images of five ovarian immature teratomas in which the solid components were small in comparison to the whole tumor. In (b–e), the solid component was too far away from the ultrasound probe for detailed assessment of its echogenicity to be possible. [file UOG-67-89-s004.png]

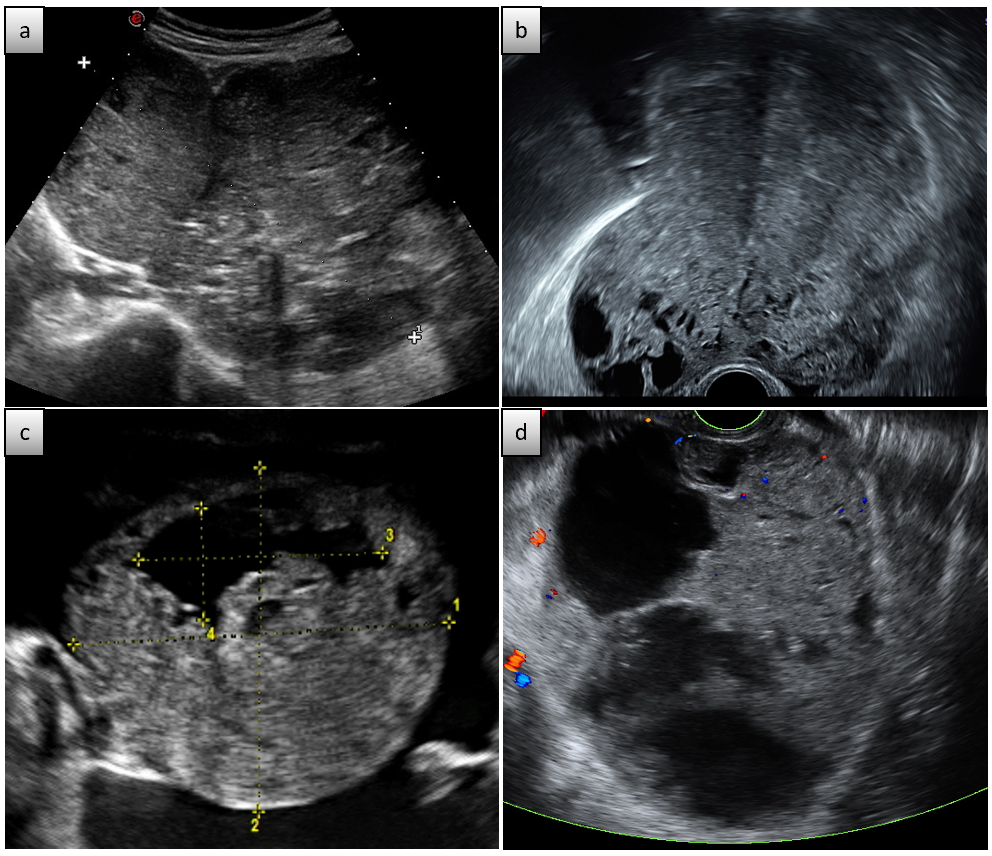

Supplement: Supplementary file 3 — Figure S3 Grayscale (a–c) and color Doppler (d) ultrasound images of four ovarian immature teratomas in which large solid components did not manifest pathognomonic echogenicity with hyperechogenic areas, cystic areas and shadowing. [file UOG-67-89-s002.png]

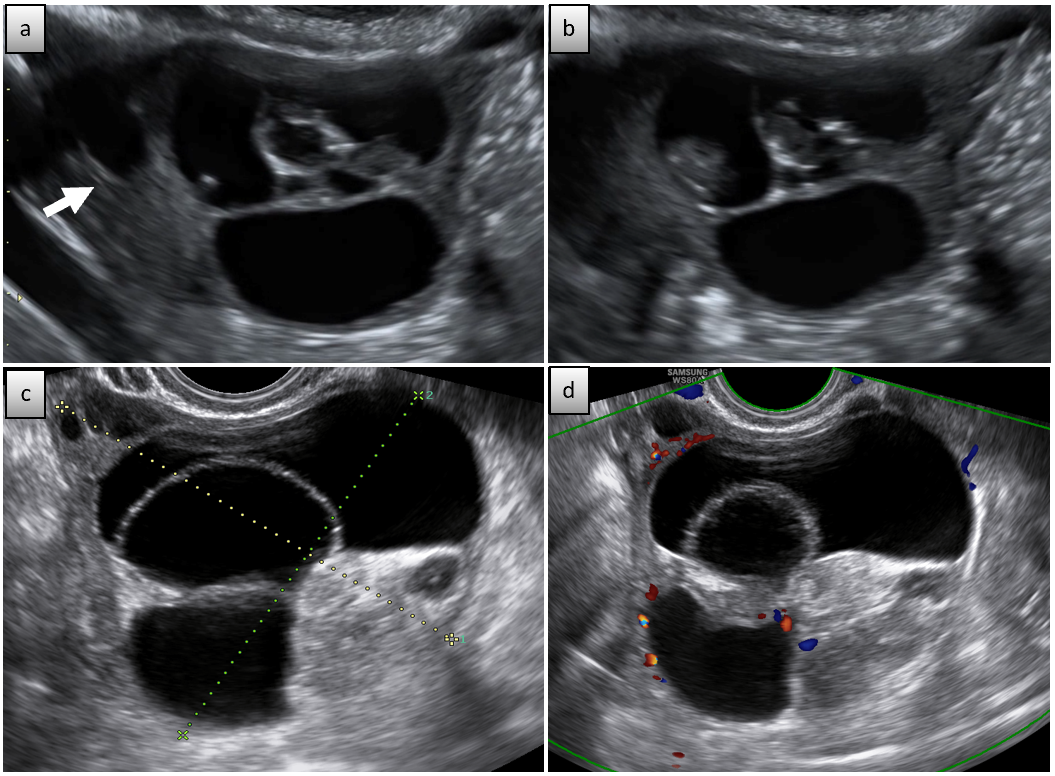

Supplement: Supplementary file 4 — Figure S4 Grayscale (a–c) and color Doppler (d) ultrasound images of two ovarian immature teratomas that did not manifest typical ultrasound features of immature teratoma. Both Stage IIA immature teratoma (largest diameter, 27 mm) (a,b) and Grade 1 Stage IC immature teratoma (largest diameter, 65 mm) (c,d) manifest normal ovarian parenchyma visible adjacent to the tumor (white arrow). Both patients had a personal history of a surgically removed malignant ovarian tumor in the contralateral ovary (borderline tumor (a,b) and yolk sac tumor (c,d), respectively). [file UOG-67-89-s001.png]

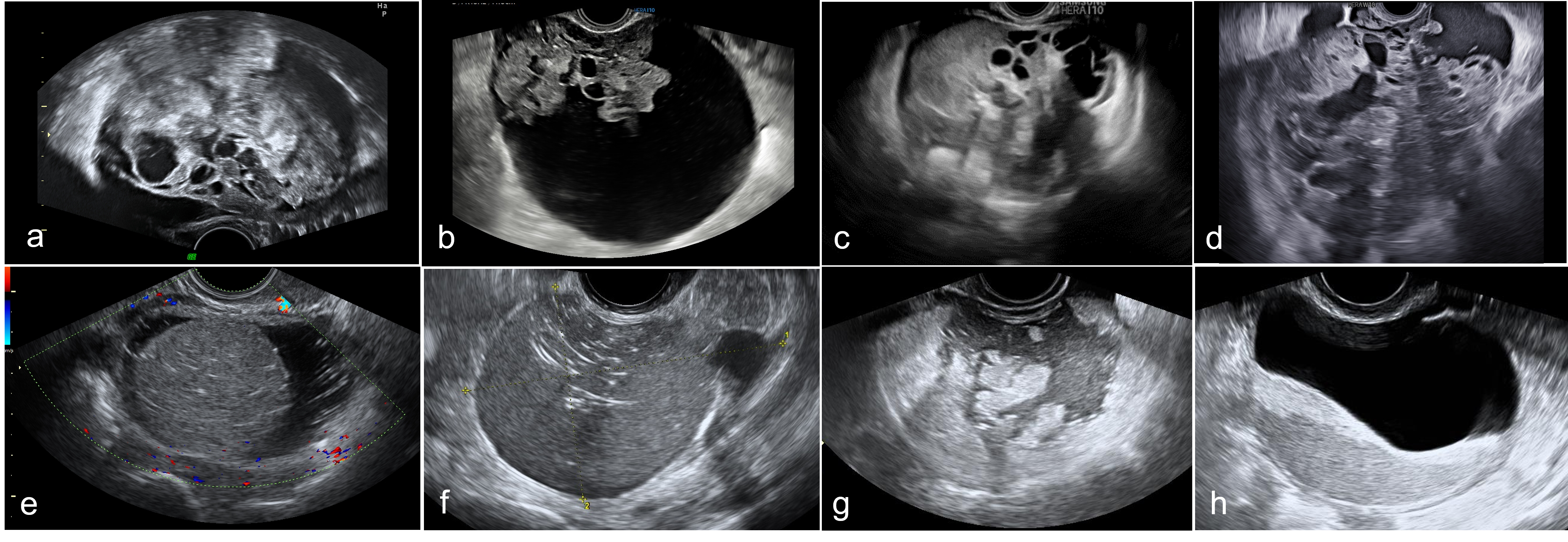

Supplement: Supplementary file 5 — Figure S5 Typical ultrasound images of immature teratomas with pathognomonic appearance of the solid components (i.e. cystic areas, hyperechogenic areas and shadowing) (a–d), contrasted with typical ultrasound images of dermoid cysts (e–h) which display features resembling ‘white ball’ (e), ‘dots and lines’ (f), ‘cotton wool tufts’ (g) and ‘mushroom cap’ (h). The ultrasound images of the dermoid cysts are reproduced from Heremans et al. 26 . [file UOG-67-89-s006.png]

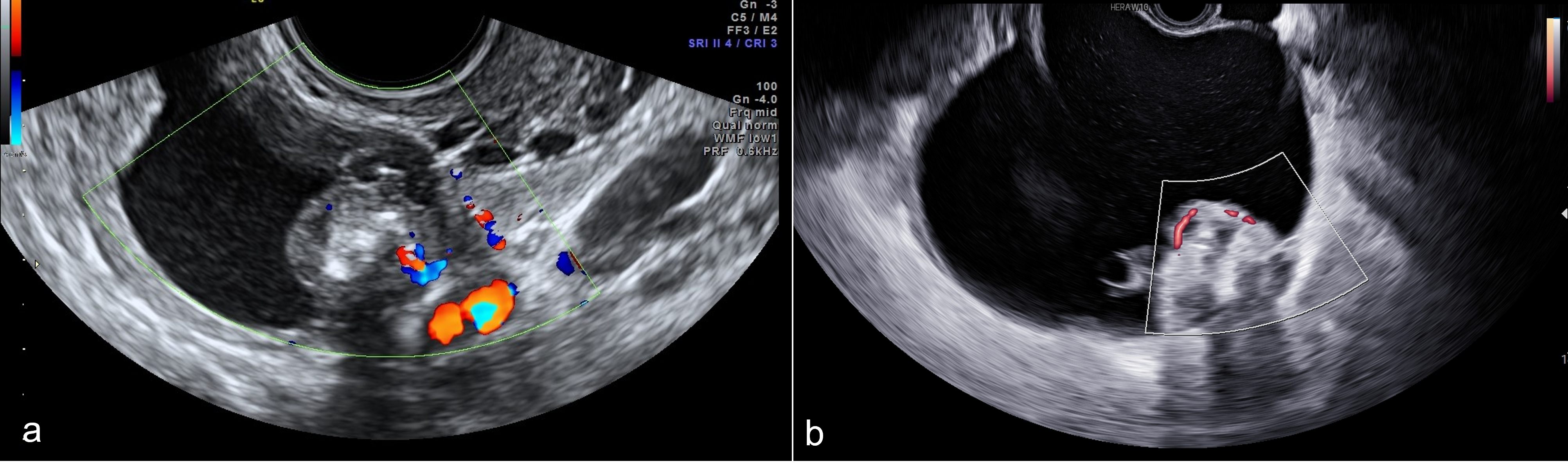

Supplement: Supplementary file 6 — Figure S6 Color (a) and power (b) Doppler ultrasound images of benign cystic teratoma with atypical ultrasound appearance (a) and an immature teratoma with only a small solid component (b). The ultrasound image of the benign cystic teratoma is reproduced from Heremans et al. 26 . [file UOG-67-89-s003.png]
