# Supplementary material for: Imaging in gynecological disease (29): clinical and ultrasound features of primary ovarian immature teratoma
Source: Ultrasound Obstet Gynecol. 2025 Oct 21;67(1):89–99. doi: 10.1002/uog.70111 (PMC12757824; doi:10.1002/uog.70111)
Supplement: Supplementary file 7 — Appendix S1 List of contributing ultrasound centers. [file UOG-67-89-s007.docx]

**Contributing Centers**

Department of Woman and Child Health, “Agostino Gemelli” Foundation University Hospital, Rome, Italy (15 cases).

Department of Gynecologic Oncology, National Cancer Institute of Milan, Milan, Italy (7 cases).

Queen Charlotte’s and Chelsea Hospital, Imperial College, London, United Kingdom (6 cases).

Department of Obstetrics and Gynecology, University Hospitals Leuven, Leuven, Belgium (6 cases).

Preventive Gynecology Unit, Division of Gynecology, European Institute of Oncology, Milan, Italy (5 cases).

Department of Gynecological Oncology, Medical University of Lublin, Lublin, Poland (4 cases).

Clinic of Obstetrics and Gynecology, University of Milan-Bicocca, San Gerardo Hospital, Monza, Italy (4 cases)

Department of Obstetrics and Gynaecology, Skåne University Hospital, Malmö, Sweden (3 cases).

Gynecology and Physiopathology of Human Reproduction Unit, S. Orsola-Malpighi Hospital of Bologna, Bologna, Italy (3 cases).

Department of Gynecological Oncology, Guy's and St Thomas' NHS Foundation Trust, St Thomas' Hospital, London, United Kingdom (2 cases).

Department of Obstetrics and Gynecology, Azienda Ospedaliero Universitaria di Cagliari, Cagliari, Italy (2 cases).

Department of Obstetrics, Gynecology, and Reproduction, Hospital Universitari Dexeus, Barcelona, Spain (2 cases)

Department of Obstetrics and Gynecology, Ziekenhuis Oost-Limburg, Genk, Belgium (1 case).

Department of General Gynecology and Gynecologic Oncology, Vienna, Austria (1 case)

Gynecological Oncology Center, Department of Obstetrics and Gynecology, First Faculty of medicine, Charles University, Prague, Czech Republic (1 case).

Department of Obstetrics and Gynecology, Erebuni Medical Center, Yerevan, Armenia (1 case)

Wright State University, Boonshoft School of Medicine, Dayton, OH, USA (1 case)
